# Factors influencing uptake of risk-reducing mastectomy among unaffected Israeli *BRCA1*/*BRCA2* pathogenic variant carriers

**DOI:** 10.1007/s10689-026-00568-x

**Published:** 2026-05-13

**Authors:** Yael Laitman, Karin Aharon, Talia Schloss, Libby Margolin, Eitan Friedman

**Affiliations:** 1 Oncogenetics Service, Assuta Medical Center, Tel Aviv, Israel; 2 Good Genes NGO, Nahariya, Israel; 3 The Gray Faculty of Medicine and Health Sciences, Tel-Aviv University, Tel Aviv, Israel

**Keywords:** *BRCA* pathogenic variant carriers, Breast cancer risk, Risk reducing mastectomy, Surveillance decisions

## Abstract

Women harboring pathogenic variant (PV) in the *BRCA1* or *BRCA2* genes (= *BRCA*) have an elevated lifetime risk for breast cancer (BC). One of the main options for active BC risk reduction is bilateral risk-reducing mastectomy (RRM). Understanding the factors influencing that decision is important for genetic-counselling and risk mitigation strategy planning. A structured questionnaire was circulated to *BRCA* carriers, members of the Good Genes NGO in Israel. Data on RRM uptake and timing, factors previously reported to be associated with decision to undergo RRM (e.g., psychosocial, family history, counselling/health-system factors) were obtained. Comparison between carriers who elected to undergo RRM with those who opted for early detection schemes were performed using logistic regression and chi square statistical analyses. Of cancer free women (*n* = 391), 272 (69.6%) elected to adhere to the recommended surveillance scheme and 119 (30.4%) elected to undergo RRM. The major reasons for electing RRM over surveillance were active BC risk reduction (4.96 ± 0.23), fear of developing BC (4.86 *±* 0.50), and having at least one relative with BC diagnosed under age 45 years. Support group discussions emerged as a stronger determinant of RRM uptake than primary care physician or religious guidance. In conclusion, among healthy Israeli *BRCA* carriers the decision to undergo RRM was influenced by a complex interplay of factors—active BC risk reduction, fear of cancer diagnosis in the context of having one relative with early onset BC and support group discussions were the major drivers of RRM in Israeli *BRCA1* carriers.

## Introduction

Women harboring pathogenic variants (PVs) in the *BRCA1* or *BRCA2* genes (= *BRCA*) are at a substantially high lifetime risks for developing breast (BC) and ovarian cancer (OvC): for BC these risks are 69–72% and for OvC 17–44% by age 80 years [1]. Female *BRCA* carriers are offered an intensified early surveillance scheme aimed at early detection of BC starting at age 25–30 years that include biannual clinical breast exam and breast imaging (MRI alternating with mammograms) [2, 3]. For active BC/ OvC risk reduction the only options are risk reducing surgeries – mastectomy (RRM) and salpingo-oophorectomy (RRSO) [4, 5]. RRM has been shown to reduce BC incidence by up to 90–95% in *BRCA* PV carriers [6]. Thus, RRM is an option that should be discussed during oncogenetic counselling of cancer-free carriers. However, the decision to undergo RRM is complex, irreversible, and involves trade-offs including surgical risks, psychosocial and body‐image consequences, and implications for breast feeding or childbearing timing, especially among young women. Despite the proven effect of RRM on active BC risk reduction, uptake of RRM among unaffected *BRCA* carriers appears to vary widely across countries and over time. Country-level analyses of more than 6,000 *BRCA* carriers demonstrated striking international heterogeneity in the uptake of RRM. In the multinational study by Metcalfe et al. [7], RRM was chosen by 49.9% of U.S. carriers, approximately 40% of U.K. carriers, and only 4.5% of Polish carriers, representing the lowest rate among the ten participating countries. Comparable figures have been reported in large national cohorts: the Danish population-based registry of 3,067 *BRCA* PV carriers showed 45–55% uptake of RRM [8], with similar rates reported by a Norwegian national series [9]. UK prospective data from Evans et al. [10] confirmed a 47.7% twenty-year uptake of RRM in *BRCA* carriers. A Czech Republic retrospective cohort [11] of 496 healthy *BRCA* PV carriers from a surveillance program (2000–2020) found RRM uptake of ~ 31.5% with a significant increase after 2013 (12% in 2005–12 vs. 31.6% in 2013–20). A recent review of 58 studies involving more than 30,000 high-risk women also confirmed wide inter-country variation [12].

Factors affecting the decision to undergo RRM have been reported to include a wide range of variables: reproductive issues, psychosocial parameters, number of previous breast biopsies, family cancer history and age at cancer diagnosis in relatives, medical and genetic counselling involvement, spouse and family support, and system-level factors (e.g., physician guidance, availability of breast reconstruction, insurance coverage of the procedure) [7, 12, 13–17]. Understanding why unaffected carriers elect or decline RRM is essential for optimizing genetic counselling, patient-centered risk communication, and shared decision-making.

Specifically in Israel, data on RRM uptake and the factors that guide this decision are sparse. A questionnaire-based, single high-risk clinic study conducted by Laitman et al. [18] among 170 cancer-free *BRCA* PV carriers reported RRM uptake of 13% (22/170) at follow-up ≥ 18 months. A prior Israeli survey (2004–06) of 99 women (43% carriers) reported ~ 19% of carriers underwent RRM, and ~ 25% had positively considered it [19]. In this manuscript we explored the factors affecting the decision to undergo RRM in cancer free Israeli *BRCA* PV carriers using a questionnaire-based data collection scheme.

## Methods

**Participants**—Israeli women who were all *BRCA* PV carriers and members of the Good Genes nonprofit organization (https://goodbrcagenes.org/) were eligible for participation. The Good Genes NGO includes carriers from diverse geographic, religious, and socioeconomic backgrounds across Israel, mitigating concerns regarding single-clinic referral bias. After an initial notification of the objectives of the study and explaining the de-identified manner of data collection and analysis, a request for consent to participate was made and obtained. Each consenting participant was sent the questionnaire and returned the filled questionnaire via a secure link. The study protocol was approved by the local Ethics committee (SMC 1492-14).

**Instrument**—Participants completed a structured questionnaire designed to assess factors influencing decisions regarding RRM in *BRCA* PV carriers. The questionnaire integrated validated domains from prior surveys addressing attitudes toward surgical risk-reduction and preventive strategies in hereditary breast–ovarian cancer syndromes [e.g., 9, 20, 21]. The parameters evaluated included: age at genotyping, mutated gene, reason for genotyping, personal and family cancer history, reproductive variables, perceived cancer risk, body-image concerns, and psychological distress [15, 22]. Additional items explored were information sources, cultural and familial influences, medical professionals’ recommendations, and religious beliefs and practices [14, 23]. Responses were recorded using 5-point Likert scales (1 = “strongly disagree” to 5 = “strongly agree”) with free-text fields for qualitative insights. The questionnaire was pilot tested in a subset of 25 carriers for clarity and internal consistency before dissemination to the full cohort.

### Statistical methods

Analyses were restricted to women with PVs in *BRCA1/BRCA2* who were cancer-free at the time of management decision. The primary comparison was between women choosing RRM and those opting to be managed with surveillance. Age at *BRCA* carrier diagnosis was treated as a continuous variable. Because age distributions were not assumed to be normal, we used the Mann–Whitney U test to compare age between RRM and surveillance groups [24]. Extreme outliers in age within the surveillance group were identified using the interquartile range (IQR) method (values < Q1 − 1.5 × IQR or > Q3 + 1.5×IQR) and excluded for age-specific descriptive statistics and the age comparison.

Family history variables were summarized as both binary indicators (any affected first- or second-degree relative; any first-degree relative with BC or OvC; any first-degree relative with BC < 45 years) and counts of affected relatives. Between-group differences in binary family history variables were evaluated using Pearson’s chi-square tests. For count variables (number of affected first- or second-degree relatives), we reported means, standard deviations, and medians by management group; where formal comparisons were performed, non-parametric tests were preferred given skewed distributions. To account for multiple comparisons across family history variables, p-values from univariate tests were adjusted using the Benjamini–Hochberg false discovery rate (FDR) procedure, and we report both raw p-values and FDR-adjusted q-values [25]. All tests were two-sided, and statistical significance was a priori defined as q < 0.05. Analyses were additionally stratified by gene (*BRCA1* vs. *BRCA2*) to explore whether patterns of age and family history differed by genotype.

We used multivariable logistic regression to estimate adjusted odds ratios for undergoing RRM Vs. surveillance, including age at *BRCA* carrier diagnosis, *BRCA1* versus *BRCA2* status, and the presence of at least one first-degree relative with BC diagnosed before age 45 years as predictors. Odds ratios were obtained by exponentiating the logistic regression coefficients, providing adjusted effect estimates for each predictor on the likelihood of choosing RRM.

Participants rated the influence of multiple decision-related factors on a 1–5 Likert scale (1 = not at all important, 5 = extremely important) [26], when choosing between RRM and continued surveillance. Items were grouped conceptually into five domains: (1) risk-perception factors (e.g. active BC risk reduction, fear of developing cancer, family history), (2) family-oriented factors (e.g. worrying about the children, not wanting to become a burden on the family), (3) treatment-burden/side-effect concerns (e.g. fear of surgical complications, fear of recovery from surgery, reducing the need for frequent surveillance, preventing frequent biopsies, worry about body image), (4) information-source factors (e.g. information from clinical teams, general internet sources, support group), and (5) religious/spiritual factors (e.g. personal religious beliefs, guidance from a spiritual leader - a rabbi). For each item, we calculated the mean and standard deviation; we then computed domain-level averages to compare the relative influence of these broader constructs.

## Results

A total of 512 single *BRCA* PV carriers agreed to participate and filled the questionnaire: 308 (60%) were *BRCA1* PV carriers and 204 (40%)—*BRCA2* PV carriers. The majority of *BRCA1* and *BRCA2* PVs reported herein (~ 85%) were one of the three predominant PVs in the Ashkenazi population—[27]. Notably, mean age (*±* SD) at data collection was 45.5 ± 10.8 years (range 29–77; median 44 years) and mean ± SD at genotyping and *BRCA* carriership disclosure 37.9 ± 10.6 (range 25–70; median 37 years). Of participants, 121 were diagnosed with cancer – 102 (84.3%) with BC. Mean age at BC diagnosis 43.5 ± 11.3 years (range 24–68; median 41 years).

Among cancer-free *BRCA* PV carriers, 119 (30%) elected RRM and 272 (70%) chose surveillance. Notably, all women opting for surveillance underwent semiannual breast imaging by MRI (alternating with mammography). The mean time from genetic test results disclosure to actual RRM was 4.27 ± 4.7 years (range 0–19; median 2 years). Women undergoing RRM were slightly older at *BRCA* carrier diagnosis than those managed with surveillance (mean 37.6 ± 8.7 vs. 35.6 ± 9.8 years; Mann–Whitney U *p* = 0.017, q = 0.259), after exclusion of age outliers in the surveillance group. Median age at *BRCA* carrier diagnosis was slightly lower for *BRCA1* PV carriers (32 years) than *BRCA2* PV carriers (37 years) in the surveillance arm, but these differences were modest and did not translate into large shifts in the relative uptake of RRM versus surveillance by gene. Family history of BC and OvC was highly prevalent in both groups, with no statistically significant differences after multiple-testing correction: ~61% of women in both RRM and surveillance groups had at least one affected first-degree relative (FDR) with BC or OvC, and ~ 38% had an affected FDR with BC. The proportion with an affected FDR with BC before age 45 was numerically higher in the RRM group (about one in five) compared with surveillance, but the difference did not retain statistical significance after FDR correction (q ≥ 0.05), although effect sizes were directionally consistent. Patterns for second-degree relatives (SDR) were similar, with overlapping distributions of the number of affected relatives in both management groups. When stratified by gene, *BRCA1* and *BRCA2* carriers showed broadly comparable family history profiles within each management strategy.

Multivariable logistic regression including age at *BRCA* carrier diagnosis, mutated *BRCA* gene, family history variables, global family history measure (any affected FDR, any affected SDR, or the number of affected relatives) was independently associated with undergoing RRM versus surveillance. However, the presence of at least one FDR with BC diagnosed *≤* 45 years of age showed the largest effect size, with higher odds of choosing RRM compared with surveillance. Among *BRCA1* PV carriers (*n* = 218), approximately one-third (32%) underwent RRM. RRM uptake was strongly associated with early-onset family history: only about one-third (32%) of *BRCA1* carriers with no FDR diagnosed with BC < 45 years chose RRM, compared with one-half (52%) of those with at least one FDR with BC diagnosed < 45 years of age. The corresponding logistic regression model showed higher predicted probabilities of RRM at younger ages and consistently higher probabilities across age for women with early-onset family history.

Among *BRCA2* carriers (*n* = 174), overall RRM uptake was slightly lower, at about one-quarter (26%). As in *BRCA1*, early-onset family history was associated with more frequent RRM: only about 25% of *BRCA2* PV carriers without an FDR with BC < 45 years underwent RRM, compared with approximately 47% of those with ≥ 1 early-onset BC in FDR. The fitted model for *BRCA2* showed the same directional pattern, with higher predicted probabilities of RRM for women with early-onset family history across the age range, although the absolute differences between groups were smaller than in *BRCA1* PV carriers.

Women opting for RRM had a different distribution of referral reasons—particularly more “family history of cancer” and less “not recorded” than the non-RRM group. This pattern strongly suggests that those choosing RRM were genotyped with clearer and more targeted indications. *BRCA1* PV carriers were more likely to undergo RRM compared with *BRCA2* PV carriers. This is clinically expected: *BRCA1* is associated with higher and earlier BC risk. Women in the RRM group were more likely to have children than those in the non-RRM group. However, the level of effect (as determined by V Cramer) was small-moderate (0.124–0.216) [28], meaning that these differences had very limited clinical implications or significance. Marital status, level of religious beliefs and practices, and parental origin of the mutation, did not differ significantly between the RRM vs. surveillance groups (Table 1).

**Table 1:** Comparison of relevant features in RRM Vs surveillance opting groups

Variable	RRM (*n* = 119)	Non-RRM (*n* = 272)	*p*-value	Cramer’s V
Age at genotyping, mean ± SD (years)	37.6 ± 8.7	35.6 ± 9.8	**0.045** ^a^	–
*Reason for genotyping*			**0.0004** ^b^	**0.216**
Screening	24	54		
Mutation in family	42	129		
Family history of cancer	52	69		
Not recorded	1	20		
*Marital status*			0.505^b^	0.059
Married	103	224		
Separated/divorced	8	28		
Single	8	20		
*Offspring*			**0.0145** ^b^	**0.124**
Yes	108	218		
No	11	54		
*Religious status*			0.497^b^	0.060
Orthodox	3	9		
Conservative	18	53		
Secular	99	210		
*Mutated gene*			**0.011** ^b^	**0.128**
BRCA1	78	139		
BRCA2	41	133		
*Parental origin of PV*			0.313^b^	0.077
Maternal	51	97		
Paternal	55	134		
Unknown	13	41		

^a^Welch’s *t*-test

^b^χ^2^ test of independence

Bold *p*-values indicate statistical significance at α = 0.05

Decision-related factors were rated on a 1–5 Likert scale, with higher scores indicating greater influence. When focusing on RRM, the most influential determinants were active BC risk reduction and fear of developing cancer, both with mean scores (4.96 ± 0.23 and 4.86 ± 0.50, respectively). Family-related motivations were also strong: “worrying about my kids” and “not to become a burden on my family” had means of 4.61 ± 1.04 and 4.08 ± 1.32, respectively, indicating that protecting children and avoiding future caregiving burden are major drivers of the choice for RRM. Additional pro-RRM factors with more than moderate influence included efforts to prevent frequent biopsies, and the wish to reduce the need for ongoing surveillance, (ranging between 3.75 and 3.77). Information from medical teams also contributed meaningfully, with average influence scores in the moderate-to-high range (3.67 ± 1.25).

Factors that tended to support continued surveillance rather than RRM were weaker in magnitude. The most prominent surveillance-leaning factors were fear of surgical complications (3.77 ± 1.3), fear of recovery from surgery (3.73 ± 1.36), and worry about body image (3.50 ± 1.39), all of which represent concerns about the immediate and long-term consequences of major surgery. Internet-based and social media information had moderate average influence (means around 3.3), but with large variability between women. The views of family and friends about surgery played a smaller, though non-negligible, role (mean ≈ 2.4). Religious beliefs and guidance from a spiritual leader (rabbi) showed consistently low mean scores (around 1.2). Overall, domain-level analyses show that risk perception and family-oriented motivations dominate the decision landscape, with treatment-burden concerns and information sources exerting moderate influence and religious/spiritual considerations contributing little on average.

Notably, women opting for RRM would make the same decision (4.76 ± 0.65), and would recommend other *BRCA* PV carriers to undergo the same risk reducing surgery (4.55 ± 0.79). Most women opting for surveillance state that they adhere to the recommended scheme (4.69 ± 0.78).

## Discussion

In the current study, the major determinants for opting to undergo RRM in Israeli cancer-free *BRCA* PV carriers were active BC risk reduction, fear of developing BC, and the wish not to become a burden on the family in case cancer develops. Additionally, family history of cancer—especially having a relative diagnosed with BC < 45 years—was associated with increased RRM uptake, primarily among *BRCA1* PV carriers genotyped between ages 30–50. Notably, the influence of family physicians and spiritual leaders was minimal.

The findings from this sizeable cohort reinforce several well-established determinants while adding granularity, particularly regarding family-history sub-patterns and the limited role of non-specialist guidance. Prospective psychological and mixed-methods studies consistently show that fear of BC, persistent cancer-related worry, and the perception that RRM offers the most definitive risk reduction strongly predict surgical decision-making. Isselhard et al. [15] and Morgan et al. [22] describe substantial emotional relief following RRM, in contrast to ongoing anxiety among women opting for surveillance. These observations align with our data, where BC risk reduction was the highest-ranked motivator and cancer worry was significantly elevated among women choosing RRM. Beyond its well-established effect on incidence reduction, emerging longitudinal data suggest that RRM may also confer a meaningful survival benefit: in large international cohorts of *BRCA1/2* carriers, BC–specific mortality following RRM is exceedingly low, with absolute risks remaining below 1% over long-term follow-up [29, 30]. Similar findings regarding the psychological drivers of RRM have been reported in German prospective cohorts [15, 31], where higher baseline anxiety and distress were independently associated with opting for RRM. In a U.S. NCI cohort, Portnoy et al. [32] showed that elevated cancer worry—particularly following false-positive screening—predicted subsequent RRM, while Padamsee et al. [33] reported that high-risk women explicitly prioritized “maximal risk reduction” and “relief from cancer fear.” Taken together, data from Germany, the United States, and Israel indicate that prevention-driven and fear-driven motivations are consistent across healthcare and cultural contexts.

Family-history intensity, including number of affected relatives and age at onset, has repeatedly been linked with RRM decisions. Gilbert et al. [34] reported higher uptake among women with multiple affected first-degree relatives, particularly when ovarian cancer occurred at a young age. Singh et al. [35] similarly showed that having relatives who died of BC significantly influenced decisions regarding risk-reducing surgery. Qualitative studies further highlight the role of “traumatic family cancer memories” as a powerful motivator [36–39], with women often describing surgery as a way to avoid repeating relatives’ experiences and reduce uncertainty. Our findings extend this literature by demonstrating that early-onset BC in a first-degree relative (< 45 years) is a particularly strong and quantifiable driver of RRM, especially among *BRCA1* PV carriers.

The increasing influence of peer support networks and BRCA “previvor” communities is also notable. In several cohorts, women engaging in in-person or online support groups reported that exposure to RRM-positive experiences helped normalize the procedure and frame it as acceptable and achievable. Bertonazzi et al. [40] reported that participants often cited peer discussions as instrumental in decision-making, while Dibble et al. [41] showed that support groups provide emotional validation and reinforce decisions. Similarly, Gaba et al. [42] demonstrated that online forums can create a strong social endorsement effect, particularly among premenopausal carriers. Morgan et al. [22] further found that support-group engagement was more common among women choosing RRM than among those opting for surveillance. Together, these findings suggest that peer narratives—both digital and in-person—not only reflect but actively shape decision-making by normalizing and supporting the choice of surgery.

In contrast, the influence of family physicians and religious leaders on RRM uptake in our cohort was minimal, consistent with prior studies. Segerer et al. [13] found no independent association between cultural or religious background and uptake of risk-reducing surgery, while Puski et al. [43] reported that BRCA carriers primarily rely on genetics specialists and close family members rather than primary-care providers. Qualitative research further supports this pattern: Graham et al. [44] described RRM as a self-directed decision, with healthcare professionals serving mainly as information providers, and Hesse-Biber and An [45] similarly found that decision-making is driven predominantly by personal risk perception, family experience, and social context. Collectively, these findings suggest that RRM decisions are embedded within lived family narratives and personal meaning rather than external authority.

Importantly, for women opting for surveillance, advances in imaging have improved the effectiveness of non-surgical strategies. MRI-based screening has been shown not only to enhance early detection and stage shift, but also to improve outcomes: Lubinski et al., reported substantial reductions in BC mortality—approaching 70–80% among *BRCA1* carriers participating in structured MRI surveillance programs—although the magnitude of benefit appears more variable in *BRCA2* carriers [46]. These data underscore that while RRM offers maximal risk reduction, modern surveillance strategies provide a meaningful alternative for selected women.

Despite these insights, several gaps remain. Future studies should focus exclusively on cancer-free BRCA carriers, excluding those already diagnosed with BC who elect contralateral RRM. Longitudinal, prospective designs with multiple time points are needed to better capture how decisions evolve. Expanding such studies to culturally diverse populations would improve generalizability, while standardized, validated questionnaires would enhance comparability across cohorts. In addition, deeper qualitative and mixed-methods approaches may help elucidate psychological factors, body-image concerns, partner and family influences, and long-term outcomes such as regret or satisfaction. Notably, although our cohort was NGO-based, uptake rates and decision patterns were comparable to population-based European cohorts, supporting external validity.

In conclusion, our data support a multifactorial model of RRM decision-making in which perceived cancer risk, family experience, emotional burden, and peer support play central roles, outweighing the influence of non-specialist external authority figures. These findings underscore the importance of incorporating psychosocial context, detailed family history, and support-network exposure into genetic counselling frameworks and decision-support tools for *BRCA* carriers.

## Data Availability

De-identified data that support the findings of this study are available from the corresponding author upon reasonable request.
